# Supplementary material for: Preterm ETs Are Significantly Reduced Compared with Adults and Partially Reduced Compared with Term Infants
Source: Children (Basel). 2022 Oct 5;9(10):1522. doi: 10.3390/children9101522 (PMC9600659; doi:10.3390/children9101522)
Supplement: Supplementary file 1 [file children-09-01522-s001.zip › children-1890751-supplementary.pdf]

Supplementary Material

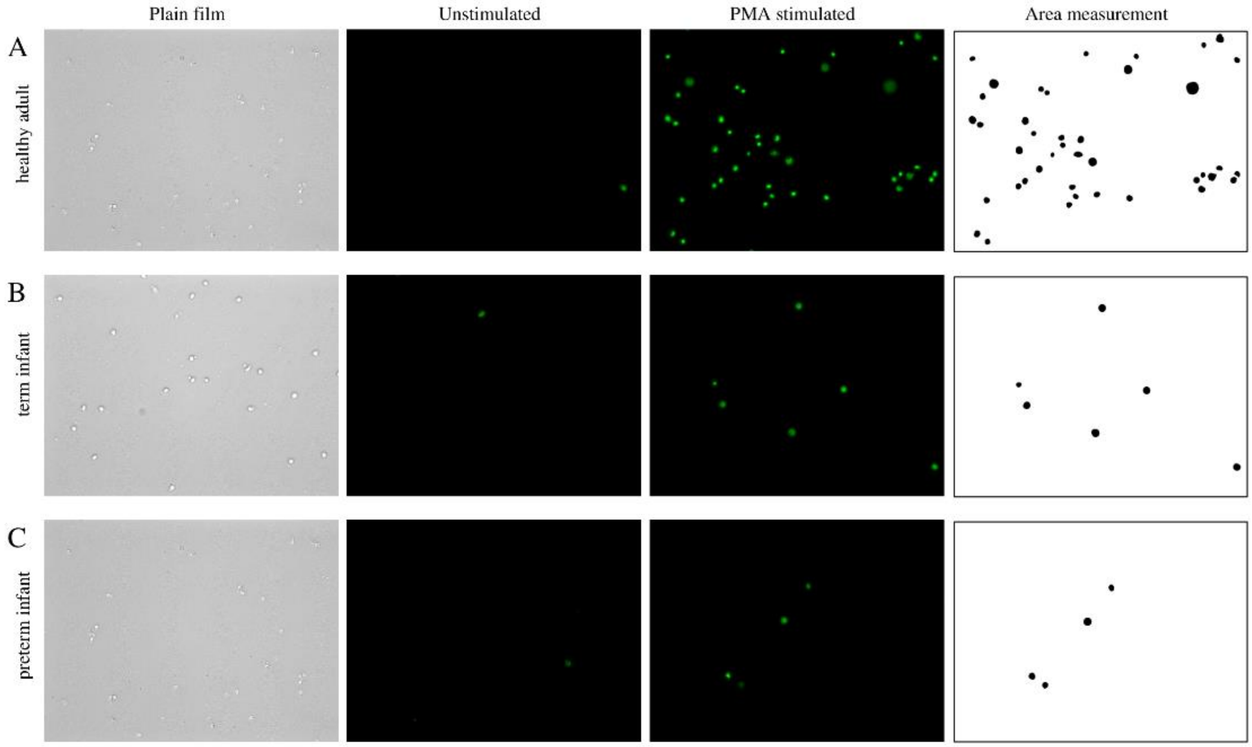

Supplementary Figure S1. METs Representative in vitro Images of METs-forming monocytes from a healthy adult (A), term infant (B), and preterm infant (C) are shown. Each row includes a plain film image, and SYTOX ® Green-stained samples without stimulation and with phorbol 12-myristate 13-acetate (PMA) stimulation. The fourth column shows edited images of the PMA-stimulated samples from which the METs area was calculated using Fiji software. Images of SYTOX ® Green-stained cells were taken using a Leica DMI 4000 B microscope with a 100 ms exposure time.

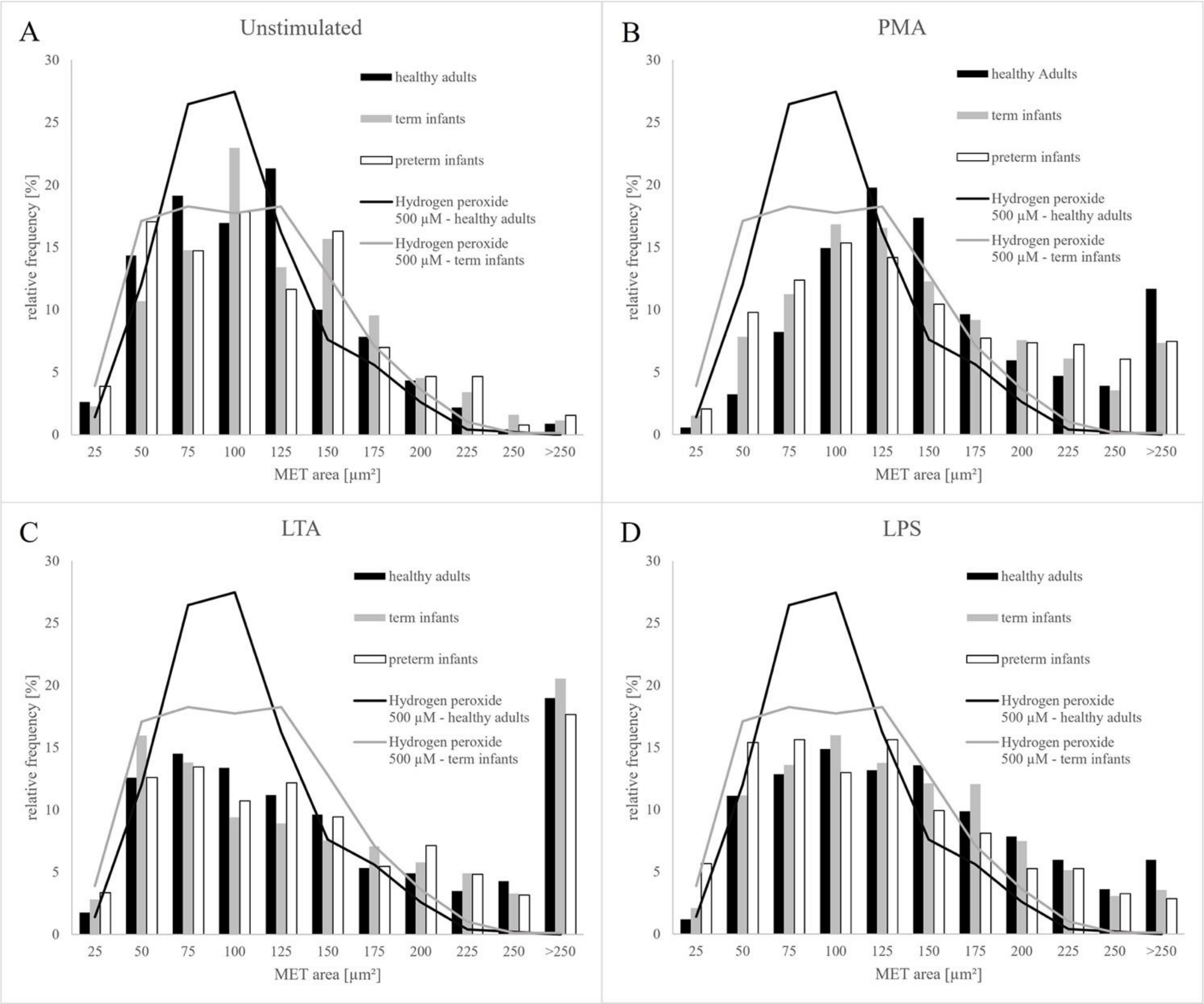

Supplementary Figure S2. METs definition by apoptosis and necrosis. Blood of healthy adults (black), umbilical blood of preterm (white) and term infants (grey) was used to isolate monocytes and induce METs. Monocytes were left either unstimulated (A) or were incubated with phorbol 12-myristate 13-acetate (PMA) (B), lipoteichoic acid (LTA) (C), or lipopolysaccharide (LPS) (D) to induce METs for 12 hours and 40 minutes. Hydrogen peroxide 500µM) was used to induce only apoptosis or necrosis to define a lower limit of METs area [µm²] for monocytes (A-D black and grey line). To calculate the METs area, we measured the fluorescent area covered by each cell using Fiji Software (Version 1.46). Due to METs formation, two peaks can be seen for the fluorescent area that is covered: The lower peak is caused by necrosis and apoptosis. The higher peak is defined as METs [μm_2]. The similar methods were established and published for NETs by other groups as well as ours [12,19].

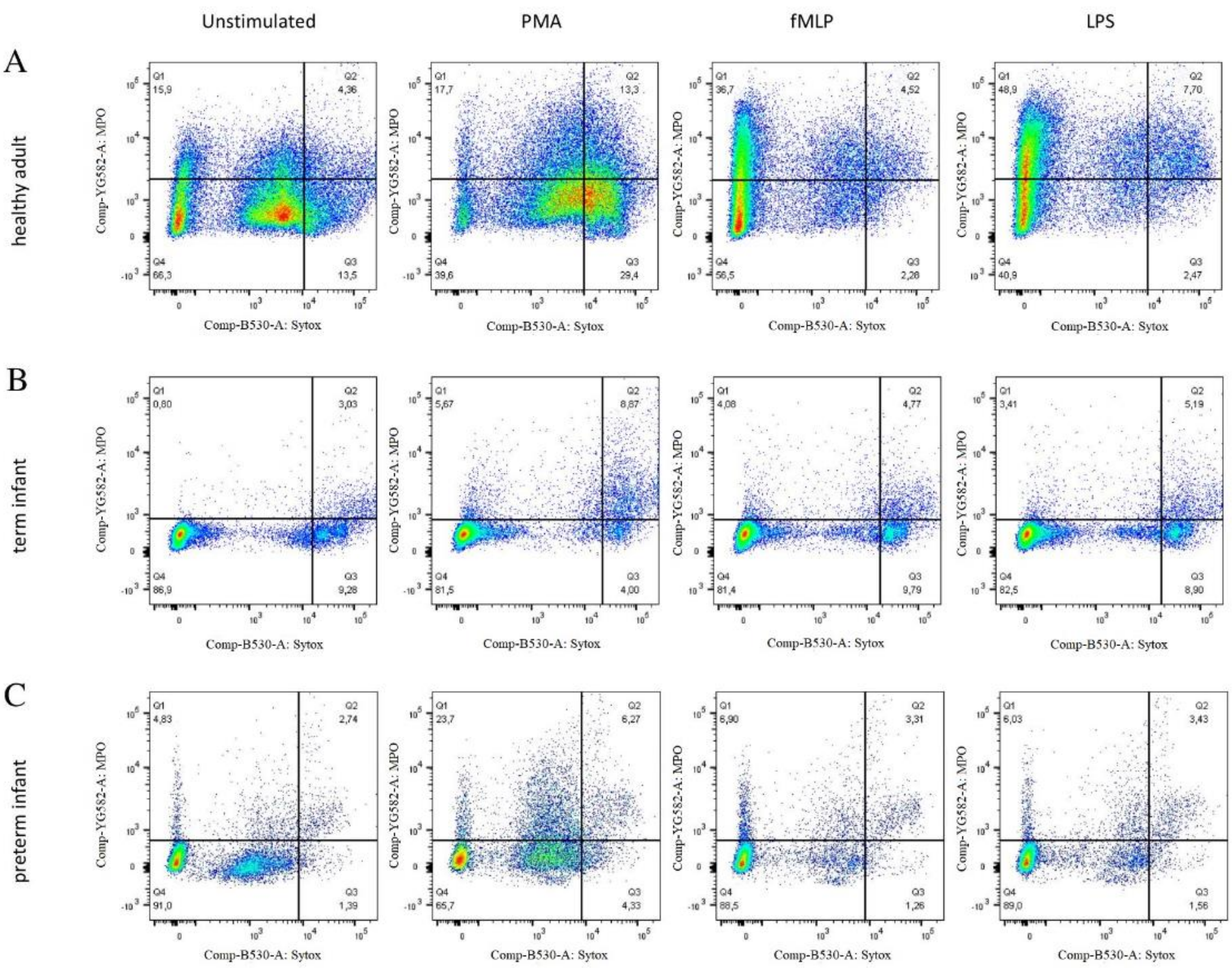

Supplementary Figure S3. Flow cytometry of NETs. NETs were analysed with LSRII. Isolated neutrophils of healthy adults (A), term infants (B) and preterm infants (C) were stimulated phorbol 12-myristate 13-acetate (PMA), N-formylmethionine-leucyl-phenylalanine (fMLP), or lipopolysaccharide (LPS) for 2 hours. Unstimulated cells were used as a control. The percentage of NETs was determined by double positive staining of MPO and SYTOX Green (quadrant Q2). To estimate the amount of MPO per NETs, which is associated with NETs as antibacterial agent, we determined the Mean Fluorescence Intensity of MPO in quadrant Q2 of all as NETs defined events.

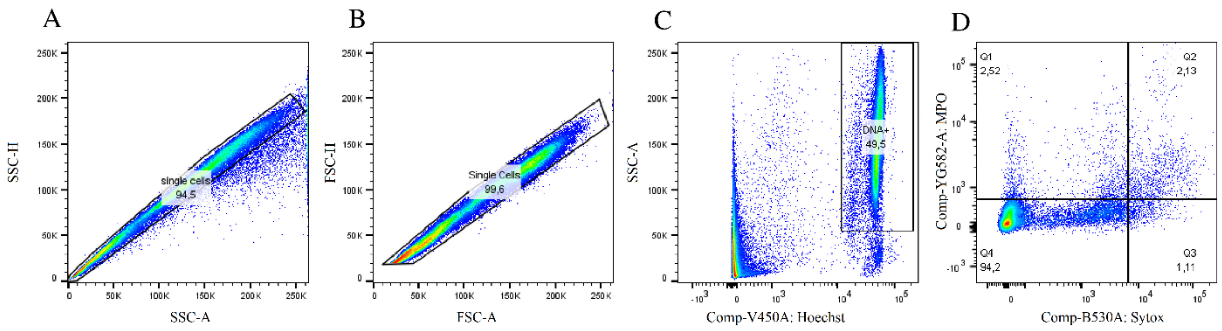

Supplementary Figure S4. Gating strategy of NET in flow cytometry. For detecting NET with flow cytometry we first discriminate doublets by SSC-A/SSC-H and FSC-A/FSC-H (A,B). To

differentiate granulocytes we stained DNA containing cells with Hoechst and measured granulocytes with SSC-A/Hoechst (C). To detect extracellular traps we used MPO and Sytox Green. Double positive events as shown in Q2 were measured as extracellular traps (D).

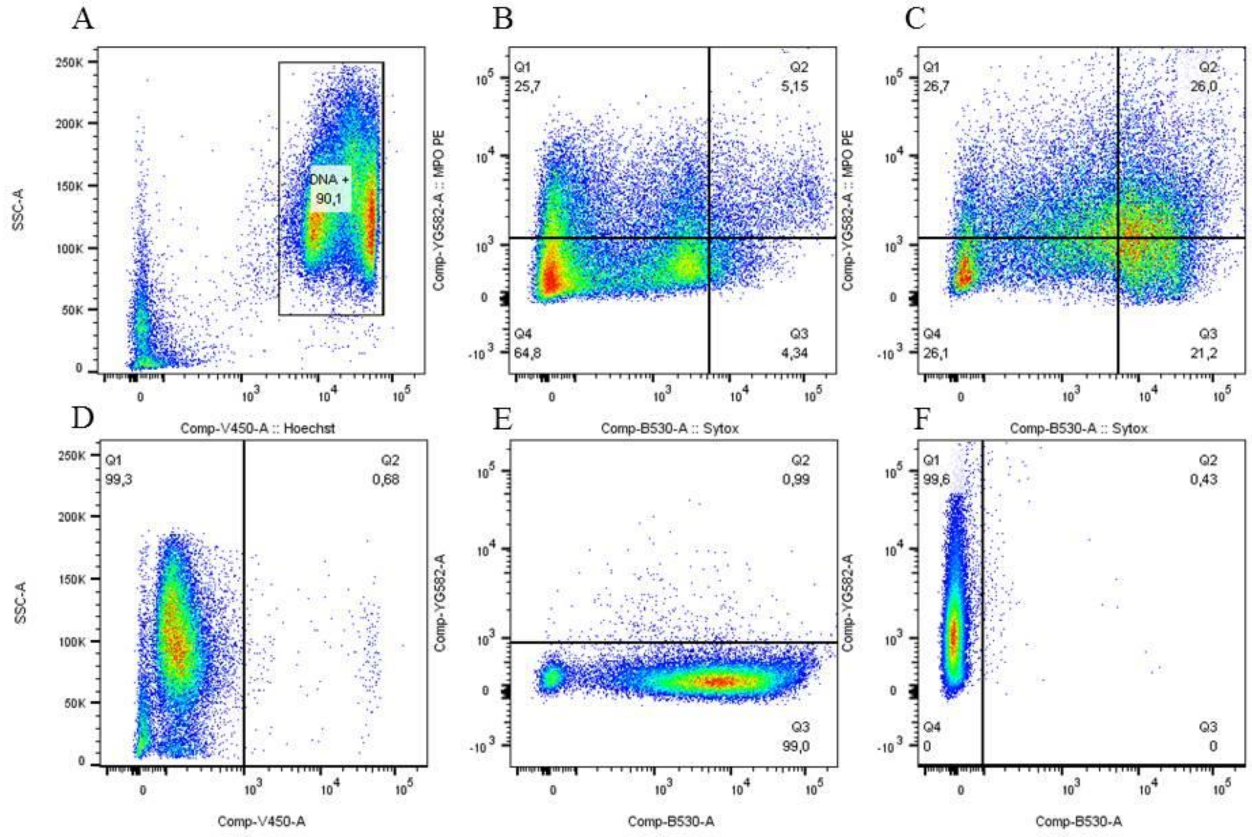

Supplementary Figure S5. Example of NETs determination by flow cytometry including FMOs. In (A) unstimulated DNA containing granulocytes are stained with Hoechst. Extracellular traps are detected as positive stained cells for MPO and Sytox as shown in (B) for unstimulated conditions and in C for PMA stimulated condition. To establish the methods, we conducted FMOs in PMA stimulated conditions for Hoechst (D), MPO (E) and Sytox (F).
In the course of our investigations, we decided to use a conservative gating strategy in which we included only cells that were still alive (HOECHST positive cells). Afterwards NETs were defined by MPO and Sytox Green-positive granulocytes. All doublets analyzed here (also compare Figure S4), were HOECHST negative and were therefore excluded. In this way, we wanted to avoid contamination by possibly necrotic cells.

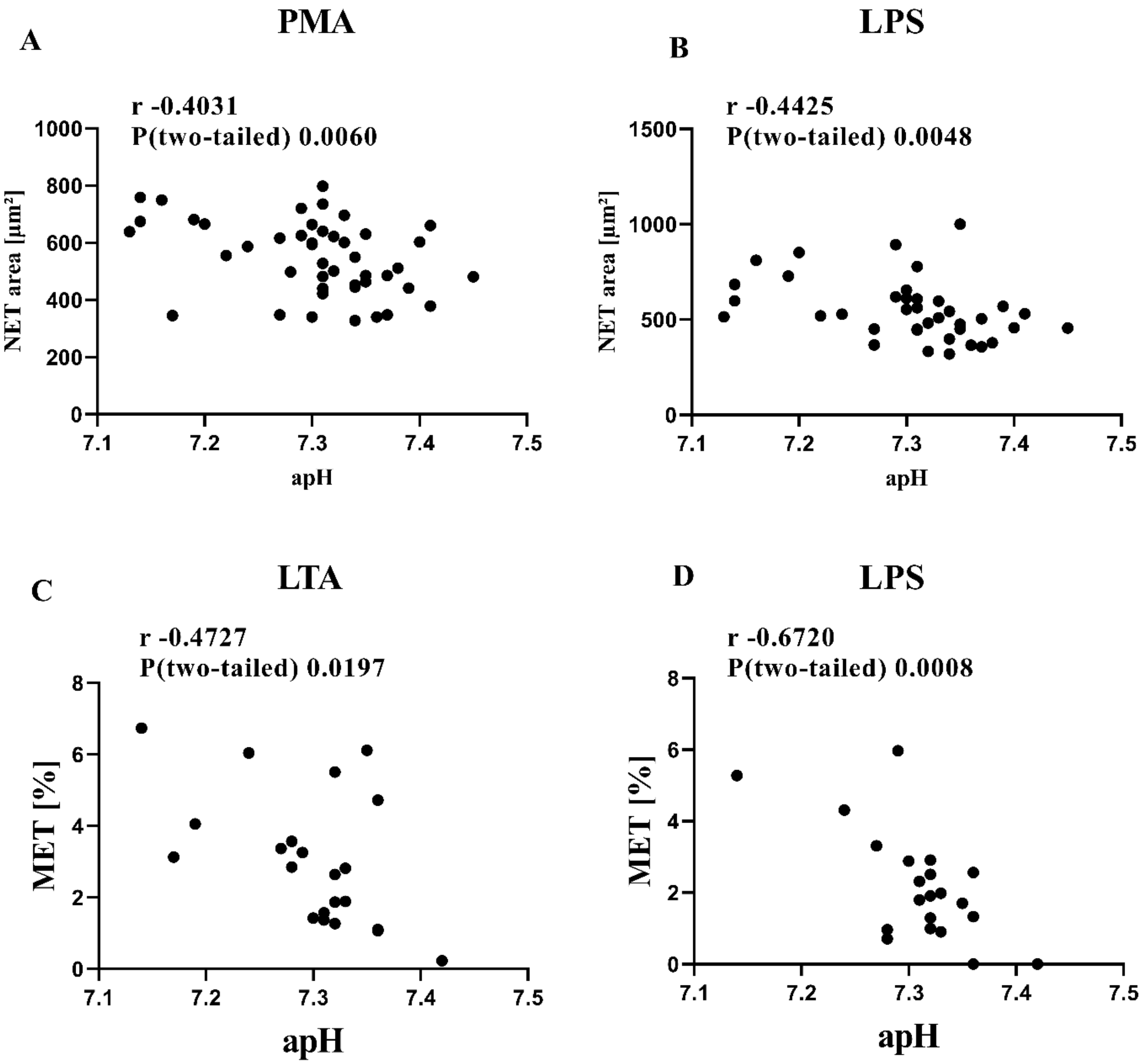

Supplementary Figure S6. Correlation of NETs and METs with arterial pH. NETs area (in µm2) (A, B) and METs percentage (%) (C, D) are illustrated for the correlation with arterial pH of umbilical blood immediately after birth. Correlation was analysed by Spearman. NETs were stimulated by PMA and LPS while METs were induced by LTA and LPS.

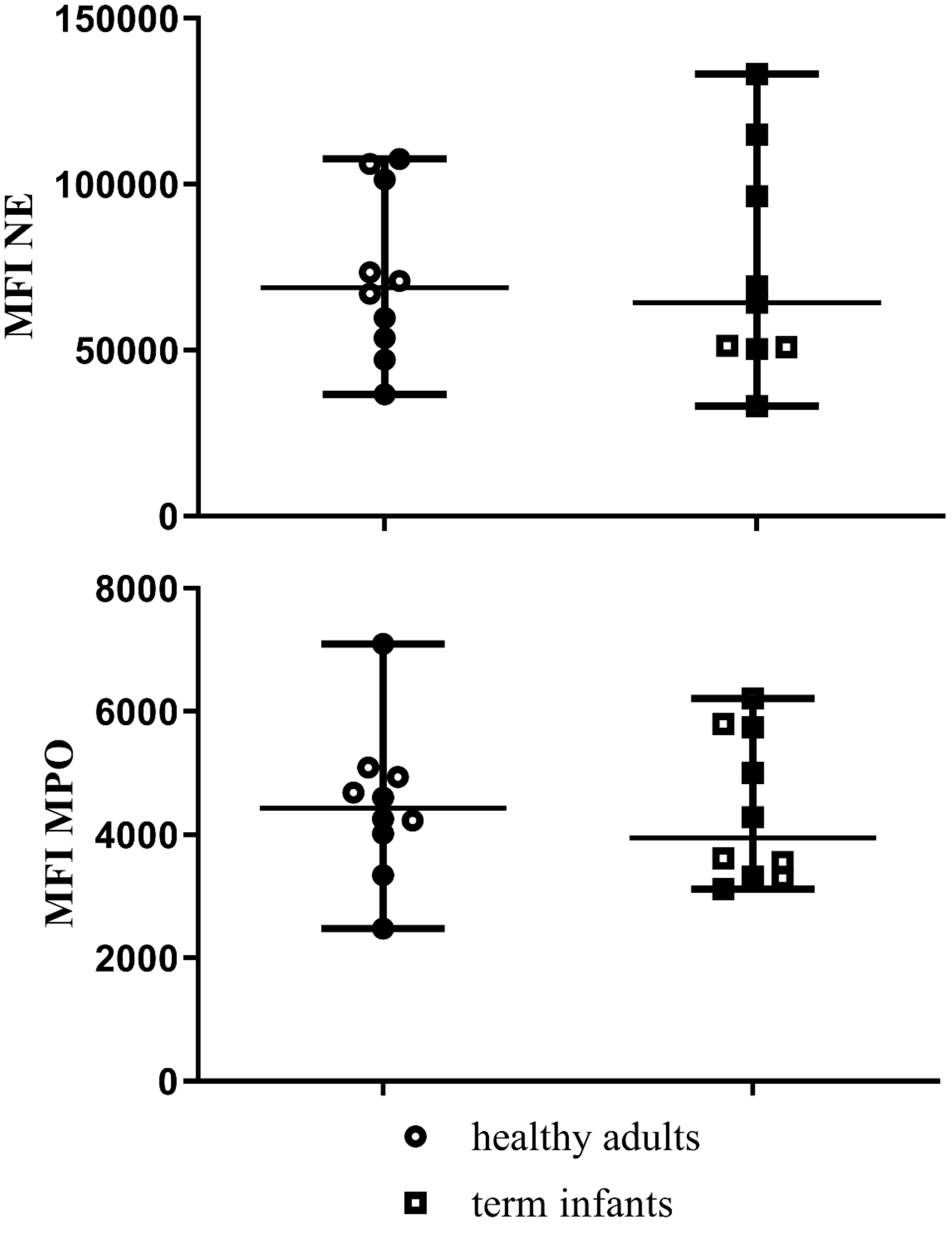

Supplementary Figure S7. The intracellular amount of NE and MPO in neutrophils of term infants. The intracellular amount (measured by Median Fluorescence Intensity) of Neutrophil elastase (NE) and Myeloperoxidase (MPO) were analysed by flow cytometry in term infants (light squares) in comparison to healthy adults (light dots). Median with interquartile range is illustrated.

	Preterm infant	Gestational age, weeks	Gender 0=male, 1=female	Birth weight, g	Small for Gestational Age 0 = no 1 = yes	Birth mode 0 = Spontaneous delivery 1 = Cesarean section	APGAR - 5 minutes	Arterial umbilical pH	Cause of preterm delivery	Parameters on admission				Early onset infection* 0=no 1=yes	Respiratory distress syndrome 0= no 1=yes	Antibiotic therapy 0= no 1=yes
										CRP mg/l	IL-6 pg/ml	Leukocytes Gpt/l	Temperature			
**NET microscopy**	1	30+1	0	1900	0	1	8	7.35	Triple I, PROM, Premature contraction	0.7	29.8	32.6	36.6	0	0	1
	2	36+3	1	2350	0	1	9	7.31	Pelvic presentation, discordant growth of children	< 3.1	< 7	< 5	36.6	0	0	0
	3	23+1	0	600	0	1	6	7.45	Pelvic presentation, PROM	< 3.1	113	< 5	37	0	0	0
	4	36+0	0	2355	0	0	10	7.22	Premature contraction	< 3.1	< 7	< 5	36.5	0	0	0
	5	29+4	0	1150	0	1	6	7.2	umbilical cord accident, PROM	< 3.1	9	16,2	36.8	0	0	1
	6	34+0	0	1970	0	1	9	7.31	PROM	< 3.1	4.7	7.5	36.5	0	0	0
	7	30+4	1	1410	0	1	8	7.31	Placental abruption	< 3.1	254	6.83	36.5	0	0	0
	8	33+2	0	1795	0	1	10	7.3	PROM	< 3.1	< 7	< 5	37.2	0	1	0
	9	30+3	0	1560	0	1	8	7.35	PROM, Premature contraction	1.7	10.4	15.3	37.1	0	0	1
	10	34+1	1	2510	0	0	9	7.29	PROM	< 3.1	< 7	< 5	38	0	0	0
	11	33+5	1	1760	0	1	8	7.4	suspected duodenal stenosis	< 3.1	< 7	< 5	36.4	0	1	0
	12	33+4	1	1970	0	1	9	7.31	Premature contraction	< 3.1	< 7	< 5	36.7	0	1	0
	13	35+0	0	2540	0	0	8	7.27	PROM	< 3.1	< 7	< 5	36.8	0	1	0
	14	29+3	0	850	1	1	10	7.35	PROM	< 3.1	< 7	< 5	37.4	0	1	0
	15	30+4	0	1820	0	1	8	7.34	Placental abruption, Placenta praevia, Vaginal bleeding, PROM	6.2	14	8.86	n.a.	0	1	0
	16	32+2	1	1970	0	1	9	7.17	Premature contraction	< 3.1	< 7	< 5	36.8	0	1	0
	17	33+6	0	3150	0	0	8	7.14	PROM, Premature contraction	< 3.1	< 7	< 5	37.2	0	1	0
	18	29+4	0	1410	0	1	8	7.3	Triple I, Vaginal bleeding, PROM	< 3.1	< 7	< 5	37.8	0	1	1
	19	34+5	0	1770	1	1	9	7.29	Placental insufficiency, pathological CTG, IUGR	< 3.1	< 7	< 5	n.a.	0	1	0
	20	29+6	1	1200	0	1	8	7.35	PROM, Premature contraction	< 3.1	10	19.8	37.5	0	1	0
	21	32+3	1	2280	0	1	9	7.38	suspected Placental abruption, PROM	< 3.1	22	< 5	37.2	0	1	0
	22	33+5	0	1780	0	1	10	7.34	IUGR	< 3.1	< 7	< 5	36.8	0	1	0
	23	36+4	0	3000	0	0	9	7.28	PROM	< 3.1	< 7	< 5	n.a.	0	0	0
	24	34+1	1	2160	0	1	10	7.3	Preeclampsia	< 3.1	< 7	< 5	37.4	0	0	0
	25	36+5	1	2360	0	1	10	7.33	Cervical insufficiency	< 3.1	< 7	< 5	n.a.	0	0	0
	26	33+5	0	1950	0	0	10	7.4	Cervical insufficiency	< 3.1	< 7	< 5	36.9	0	1	0
	27	27+6	1	1240	0	0	8	7.39	Vaginal bleeding	< 3.1	513	< 5	37.2	0	1	1
	28	32+2	1	1680	0	0	9	7.35	Cervical insufficiency, Vaginal bleeding, PROM	< 3.1	< 7	< 5	37.4	0	1	0
	29	34+1	0	2110	0	0	9	7.24	pathological CTG	< 3.1	< 7	< 5	n.a.	0	1	0
	30	36+6	1	2390	0	0	8	7.3	Triple I, Vaginal bleeding, Premature contraction	8.4	254	< 5	37.5	0	0	1
	31	28+3	1	1040	0	1	8	7.36	PROM	0.3	< 7	15.3	36.8	0	1	0
	32	33+6	1	1390	1	1	6	7.37	PROM	n.a.	702	8.1	37.1	0	1	0
	33	34+2	1	2075	0	1	9	7.32	Preeclampsia, pathological CTG	< 3.1	< 7	< 5	36.9	0	1	0
	34	31+1	0	1625	0	1	9	7.41	pathological CTG, PROM	< 3.1	< 7	< 5	37.6	0	1	0
	35	34+1	0	2335	0	0	10	7.34	PROM, Premature contraction	< 3.1	< 7	< 5	36.9	0	1	0
	36	35+6	1	2400	0	0	10	7.32	Cervical insufficiency	< 3.1	167	20.6	37	0	0	0
	37	34+4	1	2160	0	0	10	7.37	PROM	< 3.1	< 7	< 5	36.2	0	0	0
	38	35+1	1	1900	1	1	10	7.34	IUGR	< 3.1	< 7	< 5	36.7	0	1	0
	39	33+1	1	2420	0	1	9	7.39	pathological CTG	< 3.1	< 7	< 5	37.5	0	1	0
**NET FACS**	40	33+5	1	2450	0	0	9	7.33	Premature contraction	< 3.1	21	< 5	37.2	0	0	0
	41	31+0	0	1495	0	0	9	7.31	PROM	< 3.1	< 7	< 5	37.5	0	1	0
	42	32+6	1	2185	0	1	9	7.34	umbilical cord accident, PROM	< 3.1	< 7	< 5	36.9	0	1	0
	43	34+0	0	1845	0	1	9	7.29	pathological CTG, IUGR, Gestational diabetes and hypertension, Placental insufficiency	46	870	< 5	36.4	1	1	1
	44	35+2	0	2350	0	0	10	7.32	PROM	< 3.1	< 7	< 5	36.3	0	0	0
	45	34+5	1	2420	0	1	10	7.33	PROM, Premature contraction	< 3.1	< 7	< 5	37.2	0	0	0
	46	32+2	0	1480	0	1	9	7.36	Pelvic presentation, PROM	< 3.1	39	< 5	n.a.	0	1	0
	47	33+4	0	2340	0	1	8	7.35	Pelvic presentation, Premature contraction	< 3.1	< 7	< 5	37.6	0	1	0
**MET microscopy**	48	26+2	0	990	0	1	9	7.42	Premature contraction	< 3.1	< 7	< 5	n.a.	0	1	0
	49	36+1	1	2950	0	1	10	7.31	Vaginal bleeding, Placenta praevia marginalis	< 3.1	< 7	< 5	n.a.	0	0	0
	50	33+5	1	2450	0	0	9	7.33	Premature contraction	< 3.1	21	< 5	37.2	0	0	0
	51	31+0	0	1495	0	0	9	7.31	PROM	< 3.1	< 7	< 5	37.5	0	1	0
	52	30+6	0	1370	0	1	10	7.32	Vaginal bleeding, PROM	< 3.1	< 7	< 5	37.1	0	1	0
	53	32+6	0	2640	0	1	9	7.28	Vaginal bleeding, pathological CTG, PROM	< 3.1	< 7	< 5	36.5	0	1	0
	54	32+2	0	1480	0	1	9	7.36	Pelvic presentation, PROM	< 3.1	39	< 5	n.a.	0	1	0
	55	33+4	0	2340	0	1	8	7.35	Pelvic presentation, Premature contraction	< 3.1	< 7	< 5	37.6	0	1	0

PROM - premature rupture of membranes
IUGR - suspected Intrauterine growth restriction
CTG - Cardiotocography
* C-reactive protein > 10 mg/liter and symptoms of an infection during the first 72 h of life

Supplementary Table S1. Detailed characteristics of analysed preterm populations for NETs and METs assays.
